# Supplementary material for: The Systems Biology Research Tool: evolvable open-source software
Source: BMC Syst Biol. 2008 Jun 29;2:55. doi: 10.1186/1752-0509-2-55 (PMC2446383; doi:10.1186/1752-0509-2-55)
Supplement: Additional file 1 — SBRT Archive. An archive of the current version of the Systems Biology Research Tool. [file 1752-0509-2-55-S1.zip › sbrt-1.4.0/doc/developers_guide/api/sbrt/shell/mng/class-use/ShellProcessManager.html]

Uses of Interface sbrt.shell.mng.ShellProcessManager


|  |  |  |
| --- | --- | --- |
| **Overview** \| **Package** \| **Class** \| **Use** \| **Tree** \| **Deprecated** \| **Index** \| **Help** | |  |
| PREV   NEXT | **FRAMES**    **NO FRAMES**     **All Classes** |  |


---


## **Uses of Interface sbrt.shell.mng.ShellProcessManager**

| Packages that use ShellProcessManager | |
| --- | --- |
| **sbrt.shell.mng.algebra** | Provides classes for creating algebra process managers. |
| **sbrt.shell.mng.fba** | Provides classes and interfaces for creating Flux Balance Analysis process managers. |
| **sbrt.shell.mng.graph\_theory** | Provides classes and for creating graph theoretical process managers. |

Uses of ShellProcessManager in sbrt.shell.mng.algebra

| Classes in sbrt.shell.mng.algebra that implement ShellProcessManager | |
| --- | --- |
| `class` | `LinearCombConverterManager`             This class is used to convert linear combinations to matrices. |
| `class` | `MatrixConverterManager`             This class is used to convert matrices to linear combinations. |
| `class` | `MultipleVectorsConverterManager`             This class is used to convert files containing multiple vectors into a series of files, each containing a single vector. |
| `class` | `SingleVectorConverterManager`             This class is used to convert multiple files containing single vectors into a single file containing multiple vectors. |

Uses of ShellProcessManager in sbrt.shell.mng.fba

| Classes in sbrt.shell.mng.fba that implement ShellProcessManager | |
| --- | --- |
| `class` | `BiggSbmlReaderManager`             This class is used to manage the reading of SBML files obtained from the BiGG Database. |
| `class` | `BiggSbmlTranslaterManager`             This class is used to manage the translation of SBML files from the BiGG Database into the Systems Biology Research Tool's *FBA Reaction Files* and *FBA Catalyst Files*. |
| `class` | `MetatoolFileWriterManager`             This class is used to manage the writing of input files for Metatool. |
| `class` | `PalssonSbmlReaderManager`             This class is used to manage the reading of SBML files obtained from Palsson's website. |
| `class` | `PalssonSbmlTranslaterManager`             This class is used to manage the translation of SBML files from Palsson's website into the Systems Biology Research Tool's *FBA Reaction Files* and *FBA Catalyst Files*. |
| `class` | `SbmlWriterManager`             This class is used to manage the translation of Systems Biology Markup Language (SBML) files into Systems Biology Research Tool (SBRT) Fluxome Files. |
| `class` | `SimpleRxnFileTransManager`             This class is used to manage the translation of simple reaction files files into Systems Biology Research Tool (SBRT) FBA Reaction Files. |

Uses of ShellProcessManager in sbrt.shell.mng.graph\_theory

| Classes in sbrt.shell.mng.graph\_theory that implement ShellProcessManager | |
| --- | --- |
| `class` | `UniqueCycleIdManager`             This class is used to identify the unique cycles in files that contain collections of cycles. |

---


|  |  |  |
| --- | --- | --- |
| **Overview** \| **Package** \| **Class** \| **Use** \| **Tree** \| **Deprecated** \| **Index** \| **Help** | |  |
| PREV   NEXT | **FRAMES**    **NO FRAMES**     **All Classes** |  |


---
